# Surgical Outcomes and Comparative Analysis of Transduodenal Ampullectomy and Pancreaticoduodenectomy: A Single-Center Study

**DOI:** 10.1245/s10434-021-11190-9

**Published:** 2021-12-20

**Authors:** Eun-Ki Min, Seung Soo Hong, Ji Su Kim, Munseok Choi, Hyeo Seong Hwang, Chang Moo Kang, Woo Jung Lee, Dong Sup Yoon, Ho Kyoung Hwang

**Affiliations:** 1 Division of Hepatobiliary and Pancreatic Surgery, Department of Surgery, Severance Hospital, Yonsei University College of Medicine, Seodaemun-gu, Seoul 03722 South Korea; 2 Department of Surgery, Yongin Severance Hospital, Yonsei University College of Medicine, Seoul, South Korea; 3 Department of Surgery, National Health Insurance Service Ilsan Hospital, Ilsan, South Korea

## Abstract

**Background:**

Transduodenal ampullectomy (TDA) is performed for adenoma or early cancer of the ampulla of Vater (AoV). This study aimed to analyze the short- and long-term outcomes of TDA (TDA group) when compared with conventional pancreaticoduodenectomy (PD) or pylorus-preserving pancreaticoduodenectomy (PD group).

**Methods:**

Patients who underwent TDA between January 2006 and December 2019, and PD cases performed for AoV malignancy with carcinoma in-situ (Tis) (high-grade dysplasia, HGD) and T1 and T2 stage from January 2010 to December 2019 were reviewed.

**Results:**

Forty-six patients underwent TDA; 21 had a benign tumor, and 25 cases with malignant tumors were compared with PD cases (n = 133). Operation time (*p* < 0.001), estimated blood loss (*p* < 0.001), length of hospital stays (*p* = 0.003), and overall complication rate (*p* < 0.001) were lower in the TDA group than in the PD group. Lymph node metastasis rates were 14.6% in pT1 and 28.9% in pT2 patients. The 5-year disease-free survival and 5-year overall survival rates for HGD/Tis and T1 tumor between the two groups were similar (TDA group vs PD group, 72.2% vs 77.7%, *p* = 0.550; 85.6% vs 79.2%, *p* = 0.816, respectively).

**Conclusion:**

TDA accompanied with lymph node dissection is advisable in HGD/Tis and T1 AoV cancers in view of superior perioperative outcomes and similar long-term survival rates compared with PD.

**Supplementary Information:**

The online version contains supplementary material available at 10.1245/s10434-021-11190-9.

Despite being the second most common periampullary malignancy following pancreatic cancer, carcinoma of the ampulla of Vater (AoV) is a rare disease accounting for < 1% of all gastrointestinal malignancies.^1,2^ Due to the rarity of the disease, current clinical practice is based on retrospective data, lacking standardized guidelines. As with other periampullary malignancies, conventional pancreaticoduodenectomy (PD) or pylorus-preserving pancreaticoduodenectomy (PPPD) is performed most frequently for ampullary carcinoma; it includes complete resection of the primary lesion and radical lymph node dissection (LND). However, PD still causes a significant rate of complications such as postoperative pancreatic fistula (POPF), delayed gastric emptying (DGE), postoperative hemorrhage, and post-pancreatectomy diabetes mellitus.^3^ Consequently, a less invasive local resection, transduodenal ampullectomy (TDA), has been attempted for early stage cancers confined to the AoV or sphincter of Oddi (carcinoma in situ or pT1).

The concern regarding TDA as an alternative surgical technique to PD in early AoV cancer lies in the radicality of the procedure. This is determined by the status of the resection margin and lymph node (LN) metastasis, which are known prognostic factors for recurrence and long-term survival.^4–6^ Accordingly, previous studies focused on analyzing histopathological factors associated with LN metastasis^7–9^ as well as patterns of lymphatic spread^6,10^ and recurrence^5,11^ to suggest acceptable indications for TDA. However, the role of TDA in early AoV cancers remains inconclusive. Certain centers have performed TDA with LND and achieved oncologic outcomes comparable to PD in early AoV cancer.^12–14^ These studies suggest that TDA with LND might be a viable surgical treatment for patients unfit for PD. However, the extent of adequate regional LND is still unknown. Studies reporting their experience with TDA in early AoV cancer have been limited by sample size or a short duration of follow-up.

This study aimed to review and summarize the experience of TDA in a single large-volume center, focusing on whether TDA was an acceptable surgical treatment for patients with early AoV cancer. This was accomplished by comparing the clinical and oncologic outcomes between TDA and PD.

## Methods

### Patient and Data Collection

In this retrospective study, we reviewed and analyzed a prospectively maintained database of patients with AoV neoplasms who underwent TDA at Severance Hospital, Yonsei University College of Medicine, between January 2006 and December 2019. For a comparative analysis between TDA and PD, patients who underwent PD (either conventional PD or PPPD) for AoV neoplasms from January 2010 to December 2019 were identified.

Clinical and pathological characteristics such as age, sex, tumor size, pathologic T stage, LN metastasis, and differentiation were retrieved, along with data on perioperative and long-term patient outcomes. The pathologic stage was defined according to the seventh edition of the American Joint Committee on Cancer (AJCC) staging system. Low-grade dysplasia was considered benign and high-grade dysplasia was regarded as Tis according to the current World Health Organization classification. Complications were graded using Clavien-Dindo (CD) classification.^15^ CD grade ≥ III was considered severe. Clinically relevant POPF (CR-POPF) and DGE were defined and graded according to the International Study Group of Pancreatic Surgery.^16,17^ This retrospective study was approved by the Institutional Review Board (IRB) of Severance Hospital, Yonsei University College of Medicine (4-2020-1144).

### Indication and Procedure

At our institution, TDA was performed for ampullary neoplasms in the following situations: when endoscopic papillectomy (EP) was contraindicated (e.g., bile duct and/or pancreatic duct involvement); when a tumor was present at the basal or lateral margin after EP; local recurrence after EP; patients with suspiciously early AoV cancer confined to the AoV on endoscopic ultrasonography (EUS) or magnetic resonance imaging; and patients with AoV cancers not suitable for PD.

**Fig. 1:**
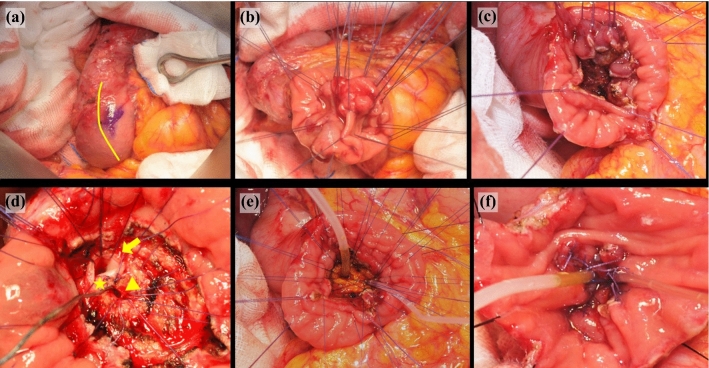
Surgical procedures of transduodenal ampullectomy. After Kocherization, the duodenum was fully mobilized from the retroperitoneum for better visualization. A 3–4 cm duodenotomy (*yellow line*) was performed longitudinally along the second portion of the duodenum (**a**). Stay sutures were applied over the incision line to maintain the opening of the duodenum. To secure a clear resection margin, mucosal stay sutures were applied on the tumor side and on the remnant mucosal side for better visualization of the resection line (**b**). Excision of the lesion was done by electrocauterization with a fine needle tip, followed by identification of the bile duct and pancreatic duct opening (**c**, **d**). For the bile duct and pancreatic duct repositioning, absorbable Vicryl® 5-0 was used. The first step was to perform three sutures on the septum between the bile duct and pancreatic duct (*star*). The next step was to make sutures with appropriate intervals along the pancreatic duct (*arrow head*) and surrounding mucosa wall. As the last step, sutures between the bile duct (*arrow*) and surrounding mucosa wall were made (**d**). Before approximating the sutured threads using a tie, short stents were inserted into the bile duct and pancreatic duct, respectively (**e**, **f**)

After laparotomy, the first step of TDA is Kocherization of the duodenum. LN 16 is dissected when malignancies are confirmed on preoperative biopsy, after EP, or if there are enlarged LNs and malignancies are suspected. LN stations 12, 13, 6, and 8 were commonly dissected when further dissection was indicated. After LND, careful palpation of the duodenum to identify the ampullary lesion was performed. A duodenotomy of 3–4 cm was performed longitudinally along the second portion of the duodenum (Fig. 1a). Stay sutures were placed over the incision line to maintain duodenal patency. To secure clear resection margins, mucosal stay sutures were placed on the tumor side as well as the remnant side for better visualization (Fig. 1b). The lesion was excised by electrocauterization with a fine needle tip, followed by identification of the bile duct and pancreatic duct opening (Fig. 1c, d). The frozen section of the deep resection margin was sent for pathological examination to ensure complete resection. For bile duct and pancreatic duct repositioning, absorbable Vicryl^®^ 5-0 was used. Three sutures were placed on the septum between the bile duct and pancreatic duct. The sutures should be placed at appropriate intervals along the pancreatic duct and surrounding mucosal wall. Finally, sutures were placed between the bile duct and surrounding mucosal wall (Fig. 1d). Before approximating the sutured threads using a tie, short stents were inserted into the bile duct and pancreatic duct (Fig. 1e). The anterior duodenal wall was closed transversely with a continuous suture using Vicryl^®^ 3-0.

### Statistical Analyses

Categorical factors are reported as frequencies with percentages, and the Pearson χ^2^ test or Fisher’s exact test was used for group comparisons. Continuous factors are presented as means and standard deviations or medians and ranges. Student’s *t*-test or Mann-Whitney *U* test was performed to analyze continuous variables. The Cox proportional hazards model was used to determine independent predictors of recurrence. Univariate analysis was performed for age, sex, tumor size, type of operation, intraoperative transfusion, T-stage, LN metastasis, lymphovascular invasion, perineural invasion, and adjuvant therapy. Variables with *p* < 0.05 were included for multivariate analysis. Disease-free survival (DFS) was defined as the time between the date of surgery and date of local or distal recurrence, and censored at last follow-up or death irrelevant to cancer recurrence. Overall survival (OS) was calculated from the date of operation to the date of death. Patients not experiencing the relevant endpoint were censored at the last follow-up. Five-year OS was calculated using the Kaplan-Meier method, and differences were examined using the log-rank test. Data analyses were performed using SPSS 25.0 software (SPSS, Chicago, IL). A *p*-value of < 0.05 was considered statistically significant.

## Results

A total of 48 patients underwent TDA for ampullary tumors at Severance Hospital from January 2006 to December 2019. One patient who underwent surgery for a combined malignancy and another who underwent PD after TDA due to R1 resection were excluded. Between January 2010 and December 2019, PD or PPPD was performed in 262 patients with AoV tumors. Excluding cases with other malignancies (n = 4), history of TDA (n = 3), M1 disease or history of preoperative chemotherapy (n = 9), combined pancreatic malignancy (n = 3), histology of adenoma/low-grade dysplasia (n = 8), T3 or T4 stage (n = 97), and neuroendocrine tumor, lymphoma (n = 5), 133 patients were included in the final PD cohort. In the PD group, 7 patients (5.3%) had HGD/Tis tumors, 47 (35.3%) had T1 tumors, and 79 (59.4%) had T2 tumors.

### Clinicopathologic Characteristics and Surgical Outcomes of TDA

Baseline characteristics and perioperative outcomes in the TDA group are summarized in Table 1. The mean age at diagnosis was 61.0 ± 9.8 years, and 25 (54.3%) were men. Sixteen (34.8%) patients underwent TDA due to endoscopic papillectomy failure, 11 for incomplete resection with margin positivity, 4 for post-endoscopic resection site tumor recurrence, and 1 for uncertain margin and the possible presence of invasive cancer.

**Table 1:** Clinicopathological characteristics and surgical outcomes in the transduodenal ampullectomy group

Characteristic	N = 46
Age (years), mean ± SD	61.0 ± 9.8
*Sex, n (%)*	
Male	25 (54.3%)
Female	21 (45.7%)
BMI (kg/m^2^), mean ± SD	24.2 ± 2.9
*ASA score, n (%)*	
1	6 (13.0%)
2	20 (43.5%)
3	13 (28.3%)
*Preoperative endoscopic resection, n (%)*	
Yes	16 (34.8%)
No	30 (65.2%)
Operation time (min), mean ± SD	218.5 ± 79.2
Estimated blood loss (ml), median (range)	50.0 (0–1000)
*Intraoperative transfusion, n (%)*	
Yes	2 (4.3%)
No	44 (95.7%)
Tumor size (mm), mean ± SD (range)	17.4 ± 7.2 (5-35)
*Operation method, n (%)*	
Open	43 (93.5%)
Laparoscopic	1 (2.2%)
Robotic	2 (4.3%)
*Diagnosis, n (%)*	
Tubular adenoma	24 (52.2%)
Tubulovillous adenoma	4 (8.7%)
Adenocarcinoma	18 (39.1%)
*Pathologic finding, n (%)*	
Benign	21 (45.7%)
Malignant (≥ HGD/Tis)	25 (54.3%)
HGD/Tis	10 (21.7%)
T1	9 (19.6%)
T2	6 (13.0%)
*Severe complication (CD grade ≥ IIIa)*	
Yes	4 (8.7%)
No	42 (91.3%)
Hospital stays (days), mean ± SD	14.9 ± 8.0

*SD*, standard deviation; *BMI*, body mass index; *ASA*, American Society of Anesthesiologists; *HGD*, high-grade dysplasia; *Tis*, carcinoma in situ; *CD*, Clavien-Dindo classification

The mean operation time was 218.5 ± 79.2 min, and the median estimated blood loss was 50.0 ml (range, 0–1000 ml). The mean tumor size was 17.4 ± 7.2 mm. Final pathology revealed 21 cases of benign neoplasm (45.7%), and the remainder were malignant. There were no cases of 90-day mortality. In-hospital complications occurred in 17 (37.0%) patients, with 4 (8.7%) cases classified as severe complications (CD grade ≥ IIIa): 3 patients underwent endoscopic intervention—1 for duodenal stricture and 2 for a distal common bile duct (CBD) stricture; 1 patient underwent reoperation due to wound dehiscence. The mean length of hospital stay was 14.9 ± 8.0 days. The readmission rate (< 90 days) was 13.0% (6 patients) due to wound oozing, minimal bile leakage, cholangitis due to CBD sludge, duodenal stricture due to inflammation, drainage site infection, and intestinal obstruction.

### Patient and Tumor Characteristics of the TDA and PD groups for Malignant AoV Tumors

Clinicopathological findings between the TDA and PD groups for malignant ampullary tumors (pHGD/Tis, T1, and T2) are summarized and compared in Table 2. There was no statistical difference between the two groups in age, sex, body mass index, American Society of Anesthesiologist score, preoperative tumor marker, and tumor size. The incidence of HGD/Tis lesion was higher in the TDA group, while a T2 lesion was more common in the PD group. LND was performed in 17 of 25 patients (68.0%) in the TDA group, with 1 case of positive LN involvement. In the PD group, the LN metastasis rate was 20.3% (27/133). Patients undergoing PD were more likely to have moderate and poorly differentiated histology (45.1% vs 24.0%, 8.3% vs 4.0%, *p* = 0.009) and showed higher rates of lymphovascular and perineural invasion (18.8% vs 8.0%, *p* < 0.001; 9.8% vs 4.0%, *p* < 0.001).

**Table 2:** Comparison of clinicopathologic findings between the transduodenal ampullectomy (TDA) and pancreaticoduodenectomy (PD) groups for malignant ampullary tumors (pHGD/Tis, T1, and T2).

Characteristic	TDA (n = 25)	PD (n = 133)	p*
Age (years), mean ± SD	61.2 ± 11.2	61.3 ± 9.9	0.918
Sex, n (%)			0.655
Male	14 (56.0%)	68 (51.1%)	
Female	11 (44.0%)	65 (48.9%)	
BMI (kg/m^2^), mean ± SD	24.1 ± 3.1	23.1 ± 2.7	0.095
*ASA score, n (%)*			0.550
1	5 (20.0%)	28 (21.1%)	
2	9 (36.0%)	61 (45.9%)	
3	11 (44.0%)	44 (33.1%)	
*Preoperative endoscopic papillectomy, n (%)*			**< 0.001**
Yes	11 (44.0%)	11 (8.3%)	
No	14 (56.0%)	122 (91.7%)	
*Preoperative tumor marker, median (range)*			
CA 19-9 (U/ml)	17.45 (1.20-346.70)	16.60 (0.10-1270.00)	0.798
CEA (ng/ml)	1.88 (0.78-8.04)	1.98 (0.48-48.20)	0.788
Tumor size (mm), mean ± SD	18.0 ± 6.5	17.8 ± 9.1	0.899
*T stage (Seventh edition of AJCC)*			**< 0.001**
pHGD/Tis	10 (40.0%)	7 (5.3%)	
pT1	9 (36.0%)	47 (35.3%)	
pT2	6 (24.0%)	79 (59.4%)	
*N stage (Seventh edition of AJCC)*			**< 0.001**
pNx	8 (32.0%)	0 (0.0%)	
pN0	16 (64.0%)	106 (79.7%)	
pN1	1 (4.0%)	27 (20.3%)	
*Stage (Seventh edition of AJCC)*			**< 0.001**
0	2 (8.0%)	3 (2.3%)	
IA	8 (32.0%)	41 (30.8%)	
IB	4 (16.0%)	58 (43.6%)	
IIB	1 (4.0%)	27 (20.3%)	
NA	10 (40.0%)	4 (3.0%)	
*Differentiation*			**0.009**
Well	11 (44.0%)	50 (37.6%)	
Moderate	6 (24.0%)	60 (45.1%)	
Poor	1 (4.0%)	11 (8.3%)	
NA	7 (28.0%)	6 (4.5%)	
*Lymphovascular invasion*			**< 0.001**
Yes	2 (8.0%)	25 (18.8%)	
No	11 (44.0%)	93 (69.9%)	
NA	12 (48.0%)	15 (11.3%)	
*Perineural invasion*			**< 0.001**
Yes	1 (4.0%)	13 (9.8%)	
No	9 (36.0%)	105 (78.9%)	
NA	15 (60.0%)	15 (11.3%)	
*Resection margin*			1.000
R0	25 (100.0%)	133 (100.0%)	
R1	0 (0.0%)	0 (0.0%)	

*SD*, standard deviation; *ASA*, American Society of Anesthesiologists; *HGD*, high-grade dysplasia; *Tis*, carcinoma in situ; *NA*, not available

^*^P-values are from the Student’s t-test or Mann-Whitney test for continuous factors, and χ^2^ (Fisher’s exact) test for categorical factors

### Perioperative Outcomes After TDA and PD for Malignant AoV Tumors

For surgical outcomes, operation time and length of hospital stay were significantly shorter in the TDA group than in the PD group (Table 3). Estimated blood loss was significantly less in the TDA group compared with that in the PD group. No patients in the TDA group required intraoperative transfusion, which was required for 10 patients (7.5%) in the PD group. The overall morbidity rate was significantly higher (74.6% vs 36.0%, *p* < 0.001) and severe complications occurred more frequently in the PD group (18.8% vs 8.0%), although statistical significance was not reached. While there was no mortality after TDA, surgical mortality occurred in 3 of 133 patients (2.3%) in the PD group due to metabolic acidosis, hypovolemic shock, and sepsis, respectively.

**Table 3:** Surgical and oncologic outcomes after transduodenal ampullectomy (TDA) and pancreaticoduodenectomy (PD) (pHGD/Tis, T1, and T2)

Outcomes	TDA (n=25)	PD (n=133)	p*
Operation time (min), mean ± SD	213.1 ± 71.8	384.4 ± 86.4	**< 0.001**
Estimated blood loss (ml), median (range)	100 (0-400)	300 (0-1900)	**< 0.001**
*Intraoperative transfusion, n (%)*			0.365
Yes	0 (0.0%)	10 (7.5%)	
No	25 (100.0%)	123 (92.5%)	
Hospital stays (days), median (range)	13 (8-37)	18 (8-99)	**0.003**
*Complications, n (%)*			**< 0.001**
Yes	9 (36.0%)	100 (75.2%)	
No	16 (64.0%)	33 (24.8%)	
*Severe complications (CD grade ≥ IIIa), n (%)*			0.376
Yes	2 (8.0%)	24 (18.0%)	
No	23 (92.0%)	109 (82.0%)	
Clinically relevant POPF, n (%)	1 (5.3%)	34 (23.6%)	0.132
Grade B	1 (5.3%)	29 (22.1%)	
Grade C	0 (0.0%)	2 (1.5%)	
DGE (grade ≥ B), n (%)	1 (4.0%)	14 (10.6%)	0.628
Duodenal stricture, n (%)	1 (4.0%)	–	
Complicated fluid collection, n (%)	0 (0.0%)	14 (10.5%)	0.128
Duodenojejunostomy anastomosis stricture, n (%)	–	2 (1.5%)	
Bleeding, n (%)	0 (0.0%)	9 (6.8%)	0.356
*Re-admission related with complications (< 90 days)*			0.226
Yes	6 (24.0%)	18 (13.7%)	
No	19 (76.0%)	118 (86.3%)	
Surgical mortality, n (%)	0 (0.0%)	3 (2.3%)	1.000
*Recurrence, n (%)*			1.000
No	20 (80.0%)	108 (81.2%)	
Yes	5 (20.0%)	25 (18.8%)	0.549
Local	0 (0.0%)	2 (8.0%)	
Systemic	2 (40.0%)	14 (56.0%)	
Both	3 (60.0%)	9 (36.0%)	
*Adjuvant therapy*			0.414
No	22 (88.0%)	105 (78.9%)	
Yes	3 (12.0%)	28 (21.1%)	

*SD*, standard deviation; *CD*, Clavien-Dindo classification.; *POPF*, postoperative pancreatic fistula; *DGE*, delayed gastric emptying

### Long-term Outcomes after TDA and PD for Malignant AoV Tumors

**Fig. 2:**
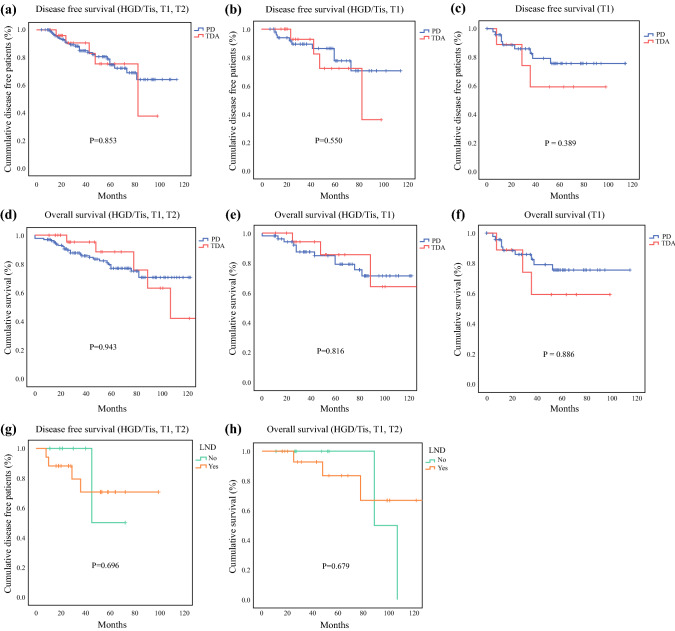
Survival outcomes of patients with malignant ampullary tumor after TDA or PD. There was no difference in 5-year disease-free survival between the TDA and PD groups regardless of whether T2 cases were included (**a**) or not (**b**). Likewise, there was no difference in overall survival rate between the two groups regardless of T2 cases (**d**, **e**). The 5-year DFS and OS of the T1 population alone were not significantly different between the TDA and PD groups (**c**, **f**). When the survival was analyzed for the TDA group focusing on whether or not LND was performed, LND did not affect 5-year disease-free survival (**g**) or overall survival (**h**). *LND*: lymph node dissection

During a median follow-up of 52 months (11–127 months) in the TDA group and 51 months (0–123) in the PD group, there were no differences in recurrence rates or patterns. Adjuvant treatment was similar between the two groups (Table 3). There was no difference in the 5-year DFS between the TDA and PD groups, regardless of whether T2 cases were included or not (Fig. 2a, b). Likewise, there was no difference in the OS rate between the two groups regardless of whether T2 cases were included or not (Fig. 2d, e). The 5-year DFS and OS of the T1 population alone were not significantly different between the TDA and PD groups (Fig. 2c, f). When the survival was further compared between subgroups of the TDA cohort stratified by the presence or absence of LND, LND did not affect either the 5-year DFS or OS (Fig. 2g, h).

The clinicopathologic findings and long-term outcomes of patients with T2 cancer who underwent TDA are summarized in Table 4. Patients 1, 2, and 3 refused subsequent PD surgery or adjuvant chemotherapy because of their comorbidity. Patients 4, 5, and 6 wanted to receive systemic chemotherapy without subsequent PD surgery. During the follow-up period, there was no disease-related death.

**Table 4:** Clinicopathologic findings of T2 patients after TDA (n = 6)

No.	Sex/Age	Comorbidity	N stage	Margin	Histologic differentiation	LVI/PNI	Adj. Tx.	Recurrence site	Time to recur (months)	Treatment for recurrence	Follow-Up period (months)	Disease-related death
1	M/82	Old age, HTN, DM, asthma, carotid artery stenosis	N0 (0/5)	R0	Moderate	No/No	–	Portocaval, aortocaval LN	10	2^nd^ Gem/Cis CTx → CTx hold	16	No (alive)
2	M/67	History of CABG, pulmonary TB, DM	Nx	R0	Well	No/UK	–	–	–	–	107	No
3	F/71	HTN, Angina	N0 (0/18)	R0	Well	UK/UK	–	–	–	–	122	No
4	M/60	Severe liver cirrhosis	Nx	R0	Moderate	No/UK	FL CCRTx	–	–	–	47	No (alive)
5	M/75	HTN	N0 (0/9)	R0	Moderate	Yes/No	5th FP CTx	–	–	–	78	No
6	M/48	DM	N0 (0/5)	R0	Well	No/UK	6th FL CTx	–	–	–	18	No (alive)

^*^P-values are from the Student’s t-test or Mann-Whitney test for continuous factors, and χ^2^ (Fisher’s exact) test for categorical factors.

*LVI/PNI*, lymphovascular invasion/perineural invasion; *Adj. Tx*.: adjuvant treatment; *HTN*, hypertension; *DM*, diabetes mellitus; *Gem*, gemcitabine; *Cis*, cisplatin; *CABG*, coronary artery bypass graft; *TB*, tuberculosis; *Nx*, lymph node dissection not done; *UK*, unknown; *FL*, 5-fluorouracil, leucovorin; *FP*, 5-fluorouracil, cisplatin

Detailed clinicopathological findings of five patients with recurrence after TDA are summarized in Supplementary Table 1. During the median follow-up period of 43 months (16–89 months), three patients died from disease progression.

### Risk Factors Affecting Recurrence

Uni- and multivariate analyses of risk factors affecting recurrence after TDA or PD are shown in Table 5. LN involvement, moderate/poor differentiation, lymphovascular invasion, perineural invasion, and adjuvant therapy were associated with disease recurrence in the univariate analysis (all *p* < 0.05). The type of operation (TDA vs PD) did not affect recurrence (*p* = 0.892). In the multivariate analysis, moderate/poor differentiation and perineural invasion remained as independent predictors of recurrence.

**Table 5:** Univariate and multivariate analysis of risk factors affecting recurrence in patients with malignant ampullary tumors (≥ pHGD/Tis) undergoing transduodenal ampullectomy (TDA) or pancreaticoduodenectomy (PD) (n = 158)

Variable	No	Univariate			Multivariate		
		HR	95% CI	p*	Exp(B)	95% CI	p*
*Age, years*							
≤ 60	68						
> 60	90	1.015	0.455–2.264	0.971			
*Sex*							
Male	82						
Female	76	1.295	0.584–2.874	0.525			
*Tumor size*							
< 18 mm	84						
≥ 18 mm	74	1.169	0.528–2.591	0.700			
*Type of resection*							
PD	133						
TDA	25	1.080	0.370–3.156	0.888			
*Intraoperative transfusion*							
No	148						
Yes	10	1.921	0.466–7.909	0.366			
*T classification*							
pHGD/Tis	17						
pT1	56	4.364	0.524–36.308	0.173			
pT2	85	4.000	0.495–32.308	0.193			
*LN metastasis*							
No	122						
Yes	28	4.632	1.870–11.475	**0.001**			
*Differentiation*							
Well	61						
Moderate/Poor	78	5.598	1.813–17.285	**0.003**	4.378	1.284–14.925	**0.018**
*Lymphovascular invasion*							
No	104						
Yes	27	4.079	1.589–10.470	**0.003**			
*Perineural invasion*							
No	114						
Yes	14	7.111	2.202–22.960	**0.001**	3.907	1.573–9.700	**0.005**
*Adjuvant therapy*							
Yes	31						
No	127	0.261	0.109–0.629	**0.003**			

*OR*, odds ratio; *CI*, confidence interval; *HGD*, high grade dysplasia; *Tis*, carcinoma in situ; *LN*, lymph node.

^*^P-values in multivariate analysis are from the Cox proportional hazard model.

## Discussion

Current invasive treatment strategies for AoV neoplasms include EP, PD, and TDA. For benign AoV adenoma or small-sized HGD/Tis lesions, EP has been gaining acceptance as the first choice of treatment if complete excision is attainable.^18–20^ However, due to the considerable rates of incomplete resection and recurrence, as well as histological upstaging after EP compared with biopsy,^19,20^ additional surgical intervention is required, especially when malignancy is confirmed. While PD remains a mainstay of treatment for AoV cancers, some studies have reported that TDA shows superior perioperative and comparable oncologic outcomes in early AoV cancer.^12,14,21^ In this study, the TDA group showed similar recurrence and 5-year survival rates, although short-term outcomes such as operation time, estimated blood loss, length of hospital stay, and overall morbidity rate were better, compared with the PD group.

Regarding surgical outcomes, TDA-associated complications such as cholangitis with CBD sludge, CBD stricture, and duodenal stricture requiring endoscopic intervention developed in a minor portion of patients (3 of 46); for cholangitis with CBD sludge and CBD stricture, endoscopic retrograde cholangiopancreatography with endoscopic retrograde biliary drainage insertion was performed. In the early period of TDA procedures, a monofilament, non-absorbable suture material was used for repositioning the bile and pancreatic ducts. Possibly, cholangitis was caused by the formation of bile sludge around the non-absorbed suture material. After changing to an absorbable material, this problem did not reoccur (Supplementary Fig. 2). Duodenal stricture occurred once using robotic TDA, but never in open TDA.

Prior studies exploring treatment of early AoV cancer have supported TDA, depending on the depth of invasion,^7,8,14,21–24^ size of tumor,^7,14,23,24^ histologic features,^8,12,23,24^ and LN metastasis.^12,14,23^ Regarding the depth of invasion, it is generally accepted that if the pHGD/Tis lesion is not amenable to EP or shows failure after EP, TDA without LND can be a feasible option, provided there is no LN metastasis from the tumor. In this study, there were 17 pHGD/Tis patients (10 in the TDA and 7 in the PD group). Among the patients who underwent LND (n = 11), there was no LN metastasis.

Extended application of TDA becomes controversial mainly due to the risks of LN metastasis, reported to be as high as 8–45.5% even in T1 ampullary cancer.^25,26^ This rate increases to 11.3–51% in T2 ampullary cancer,^7,9,11,12,25^ along with the issue of securing adequate resection margins. In the PD group where LND was performed, LN metastasis rates were 14.6% (7/48) in pT1 and 28.9% (24/83) in pT2 patients, similar to previous studies. Similarly, Beger et al. insisted that for patients with pT1 cancer, ampullectomy should be combined with local LND, involving anterior and posterior LNs of the pancreatic head and supraduodenal LNs along the foramen of Winslow.^12^ Amini et al. reported 20 cases of ampullectomy with regional lymphadenectomy in T1 ampullary cancer; regional lymphadenectomy improved survival, compared with local ampullectomy alone. This was comparable to the survival rates of patients undergoing PD.^13^ In our study, we performed regional LND in 13 of 15 patients (pT1 and pT2) in the TDA group, with a mean retrieved LN number of 10.4 (1–24). There was only one case with positive LN metastasis among these 13 patients, which may have contributed to a comparable oncologic outcome in the TDA group. Though the curative effect of regional LND was not delineated in this study due to the small sample size, considering the pattern of LN metastasis in ampullary cancer, regional lymphadenectomy of the pancreaticoduodenal LNs (#13, #17) and possibly the superior mesenteric LN (#14) appears necessary when TDA is performed in T1 ampullary cancer.^10^ As earlier studies analyzing LN metastasis patterns included all stages of ampullary cancer,^6,10^ further analysis on the pattern of LN metastasis in early ampullary cancer is needed to define the proper extent of LND during TDA.

Although the estimated 5-year survival rates for AoV cancer with pHGD/Tis or T1 and T2 were not different between the TDA and PD groups in this study, T2 ampullary cancer cannot be an adequate indication for TDA due to the high LN metastasis rate. Considering the extent of surgical dissection during the procedure, PD ensures more complete and extensive retrieval of LNs. In addition, as a technical and anatomical issue, it is usually harder to achieve an adequate safe resection margin when the cancer invades the duodenal wall, often requiring conversion to PD. In this study, all six patients of the TDA group with T2 stage refused the subsequent PD surgery, choosing close follow-up or systemic chemotherapy alone.

It should be noted that it is difficult to determine the appropriate indication for TDA for early ampullary carcinoma based on the preoperative T stage, due to the discrepancy between the preoperative biopsy and permanent pathologic stage after TDA. As shown in Supplementary Table 2, histologic upstaging took place for 14 of 25 patients (56%) after TDA compared with endoscopic biopsy. Among patients undergoing endoscopic papillectomy (n = 11), histologic upstaging occurred for two patients (18%, pT1→pT2) following TDA. In addition, although EUS is recommended for patients with HGD/Tis revealed on biopsy,^27^ the diagnostic accuracy of EUS in T staging is relatively lower in pT1 and pT2 (62% and 45%, respectively), compared with pT3–4 (88%).^28^ Since accurate T stage for early ampullary carcinoma before surgery is difficult to determine through preoperative imaging, endoscopic biopsy or papillectomy, and even during the TDA procedure, it should be mandatory during the TDA procedure to check the resection margin status by intraoperative frozen pathology. Whenever the extent of cancer requires PD, conversion should take place unless TDA is being performed on a palliative basis.

Previous studies have reported histological differentiation as one of the prognostic factors for recurrence and long-term survival.^5,9,11,12^ While histological differentiation has been determined to strongly associate with node positivity,^13^ we could only identify the histological grade and perineural invasion as independent risk factors of disease recurrence in early ampullary cancer (including T2 cancer in this study), outweighing LN metastasis. This emphasizes the significance of biological factors that lead to invasion and disease progression in early ampullary cancer after surgery. Perineural invasion has been suggested as an independent prognostic factor of survival after surgical resection of ampullary adenocarcinoma.^4,29–31^ Sudo et al. suggested that ampullary carcinoma with perineural invasion may have a prognosis similar to pancreatic adenocarcinoma and that adjuvant therapy in these cases should be assessed as a treatment strategy for improving survival.^30^ Analyzing the patterns of failure after surgical resection of early stage ampullary adenocarcinoma, Jim et al. reported a higher rate of locoregional failure for the surgery-only group as compared with patients undergoing combined adjuvant chemotherapy or radiotherapy.^32^ However, not receiving chemotherapy was not a factor predicting recurrence in the multivariate analysis in our work. When considering the recurrence patterns of a disease that appears more systemic than locoregional, the role of adjuvant chemotherapy after TDA should be explored in future studies.

There are some limitations to this study. First, the number of cases in the TDA group was low. Given the rarity of the disease, in 14 years, only 25 of 46 TDA cases with malignant ampullary tumors were suitable for a comparative study. This number was not statistically sufficient for conventional propensity score matching. Also, due to the small number of TDA cases, the study period of TDA cases was different from that of the PD group, which may have influenced long-term survival outcomes. The median follow-up time (months) for DFS and OS showed no difference between the TDA and PD groups. Second, although no difference was observed in the 5-year DFS and OS between the TDA and PD groups, comparable oncologic outcomes of the TDA group may have been due to the higher proportion of moderate/poor differentiation and positive perineural invasion in the PD group. Regarding the application of TDA to the T1 population except for HGD/Tis, although the 5-year DFS and OS were not significantly different between the TDA and PD groups, the number of patients was insufficient to conclude that TDA is applicable to the T1 stage of AoV cancer because only nine patients in the TDA group were included. These results should be validated in a large cohort study utilizing statistical techniques such as propensity score matching. Third, the surgical technique for TDA, especially regarding the extent of LND, was not completely standardized, albeit in a single-center setting. Considering that the LN metastasis rates were 14.6% at pT1 in this study, LND appears mandatory during a TDA to treat early AoV cancer. A well-designed study mapping LND is needed to define the proper extent of LND during TDA. Fourth, all pathological findings were based on the seventh edition of the AJCC; the newer eighth edition of the AJCC is not reflected in this study.

In conclusion, the results of this study suggest that TDA can be a feasible option for patients with early AoV cancer in view of superior perioperative outcomes and comparable long-term survival rates as compared with PD. TDA should be accompanied by LND, and more research is needed to determine the proper range of LND. For patients at T1 stage with risk factors such as perineural invasion and moderate/poor histologic findings, closer follow-up is recommended owing to the risk of recurrence after surgery.

## Supplementary Information

Below is the link to the electronic supplementary material.

Supplementary file1 (DOCX 17 KB)
