# Supplementary material for: Surgical Outcomes and Comparative Analysis of Transduodenal Ampullectomy and Pancreaticoduodenectomy: A Single-Center Study
Source: Ann Surg Oncol. 2021 Dec 20;29(4):2429–40. doi: 10.1245/s10434-021-11190-9 (PMC8933369; doi:10.1245/s10434-021-11190-9)
Supplement: Supplementary file 1 — Supplementary file1 (DOCX 17 KB) [file 10434_2021_11190_MOESM1_ESM.docx]

**Supplementary Table 1.** Clinicopathologic findings of patients with recurrence after TDA (n=5)

| **No.** | **Sex/Age** | **Tumor**  **Size**  **(mm)** | **T stage** | **N stage** | **Margin** | **Adjuvant treatment** | **Recurrence site** | **Time to recur**  **(Month)** | **Treatment for recurrence** | **Follow-Up period (Month)** | **Disease-related death** |
| --- | --- | --- | --- | --- | --- | --- | --- | --- | --- | --- | --- |
| 1 | M/51 | 30 | HGD | Nx | R0 | - | Local, aortocaval LN, liver | 45 | 3^rd^ FL CTx  → PPPD | 89 | Yes |
| 2 | M/62 | 15 | T1 | N0  (0/15) | R0 | - | Local and liver | 29 | 5^th^ FP CTx  → 5^th^ XELOX CTx | 48 | Yes |
| 3 | F/75 | 21 | T1 | N0  (0/14) | R0 | - | Liver | 8 | 3^rd^ Gem/Cis CTx → XELOX | 25 | Yes |
| 4 | F/63 | 12 | T1 | N1  (2/14) | R0 | 4^th^ FL CTx | Pelvic bone, retroperitoneal LN | 36 | Gem/Cis CTx with palliative RTx | 43 | No (alive) |
| 5 | M/82 | 21 | T2 | N0  (0/5) | R0 | - | Portocaval, aortocaval LN | 10 | 2^nd^ Gem/Cis CTx → CTx hold | 16 | No (alive) |

FL, 5-fluorouracil, leucovorin; PPPD, pylorus-preserving pancreaticoduodenectomy; FP, 5-fluorouracil, cisplatin; XELOX, capecitabine+ oxaliplatin; Gem, gemcitabine; Cis, cisplatin

**Supplementary Table 2.** The discrepancy between preoperative and permanent pathologic stage after TDA.

| **Preoperative diagnosis** | | | **Final pathology after TDA** | | |
| --- | --- | --- | --- | --- | --- |
| **Endoscopic biopsy (n=25)** | **Endoscopic resection (n=11)** | | **HGD/pTis** | **pT1** | **pT2** |
| LGD (n=9) | HGD/pTis | (n=5) | 5 | - | - |
|  | pT1 | (n=2) | - | 2 | - |
|  | Not done | (n=2) | 2 | - | - |
| HGD (n=7) | pT1 | (n=2) | - | 1 | 1 |
|  | Not done | (n=5) | 2 | 3 | - |
| Carcinoma (n=9) | pTis | (n=1) | 1 | - | - |
|  | pT1 | (n=1) | - | - | 1 |
|  | Not done | (n=7) | - | 3 | 4 |
| Total (n=25) |  | | 10 | 9 | 6 |
